# SAMHD1 expression modulates innate immune activation and correlates with ovarian cancer prognosis

**DOI:** 10.3389/fimmu.2023.1112761

**Published:** 2023-02-09

**Authors:** Lucía Gutiérrez-Chamorro, Eudald Felip, Adrià Bernat-Peguera, Ifeanyi Jude Ezeonwumelu, Iris Teruel, Anna Martínez-Cardús, Bonaventura Clotet, Eva Riveira-Muñoz, Margarita Romeo, Mireia Margelí, Ester Ballana

**Affiliations:** ^1^ IrsiCaixa AIDS Research Institute – and Health Research Institute Germans Trias i Pujol (IGTP), Hospital Germans Trias i Pujol, Universitat Autònoma de Barcelona, Badalona, Spain; ^2^ Medical Oncology Department, Catalan Institute of Oncology (ICO), B-ARGO (Badalona Applied Research Group in Oncology), Health Research Institute Germans Trias i Pujol (IGTP), Universitat Autònoma de Barcelona, Badalona, Spain; ^3^ Consorcio Centro de Investigación Biomédica en Red de Enfermedades Infecciosas (CIBERINFEC), Instituto de Salud Carlos III, Madrid, Spain

**Keywords:** SAMHD1, ovarian cancer, RLR (RIG-I like receptors), interferon, inflammation

## Abstract

**Purpose:**

SAMHD1 is a deoxynucleotide triphosphate (dNTP) triphosphohydrolase which has been proposed as a putative prognostic factor in haematological cancers and certain solid tumours, although with controversial data. Here, we evaluate SAMHD1 function in ovarian cancer, both *in vitro* and in ovarian cancer patients.

**Methods:**

SAMHD1 expression was downregulated in ovarian cancer cell lines OVCAR3 and SKOV3 by RNA interference. Gene and protein expression changes in immune signalling pathways were assessed. SAMHD1 expression in ovarian cancer patients was evaluated by immunohistochemistry and survival analysis was performed according to SAMHD1 expression.

**Results:**

SAMHD1 knockdown induced a significant upregulation of proinflammatory cytokines concomitant to increased expression of the main RNA-sensors, MDA5 and RIG-I, and interferon-stimulated genes, supporting the idea that the absence of SAMHD1 promotes innate immune activation *in vitro*. To assess the contribution of SAMHD1 in ovarian cancer patients, tumours were stratified in SAMHD1-low and SAMHD1-high expressing tumours, resulting in significantly shorter progression free survival (PFS) and overall survival (OS) in SAMHD1-high expression subgroup (*p*=0.01 and 0.04, respectively).

**Conclusions:**

SAMHD1 depletion correlates with increased innate immune cell signalling in ovarian cancer cells. In clinical samples, SAMHD1-low expressing tumors showed increased progression free survival and overall survival irrespective of BRCA mutation status. These results point towards SAMHD1 modulation as a new therapeutic strategy, able to enhance innate immune activation directly in tumour cells, leading to improved prognosis in ovarian cancer.

## Introduction

Ovarian cancer is the most lethal gynaecologic cancer and long term outcomes are still unsatisfactory, irrespectively of the advent of new treatment strategies (1). At present, it is clear that genetic alterations such as mutations in *BRCA1* and *BRCA2* tumour suppressor genes and other alterations in DNA repair machinery influence ovarian cancer development in a significant proportion of cases (2). In this context, the introduction of poly (ADP-ribose) polymerase inhibitors (PARPi) represented an important therapeutic step forward particularly, but not only, for BRCA mutated ovarian cancer patients (3). Furthermore, the function of the immune system has become a matter of extensive research in ovarian cancer, prompting the development of different immunotherapeutic approaches as putative effective treatments for ovarian cancer. Immunotherapy in ovarian cancers aims at the stimulation of antigen-presenting cells, the induction of antitumor immunity and the attenuation of immunosuppressive microenvironment, albeit with inconsistent results in clinical trials (4). At the same time, new insights on the complex interaction between the immune system and tumour cells in the tumour microenvironment (TME) have emerged, suggesting that ovarian cancer initiation and development as well as immune infiltration into the tumour may be influenced by a complex chemokine-signalling network (5, 6). However, the factors leading to this anti-tumor immune activation are still under investigation. Some recent works show that damaged cancer cells can induce anti-tumoral innate immunity by releasing nucleic acids that are detected by nucleic acid-sensing receptors (7). In addition, it has also been described that cytosolic DNA sensing and subsequent stimulation of innate immunity might represent a relevant pathway in anti-tumoral immune response through type I interferon (IFN) production (8). Thus, a better understanding of nucleic acid sensing pathways, including upstream and downstream regulators, is key for a better understanding of ovarian cancer immunity, the identification of novel putative therapeutic targets and the development of new potential therapeutic approaches.

SAMHD1 (sterile alpha motif and histidine/aspartic acid domain-containing protein 1) is a cellular deoxynucleotide (dNTP) triphosphohydrolase that has been recently linked to tumour initiation and development, although with controversial findings, either being recognized as a tumour suppressor in haematological cancers (9, 10) or tumour promoting in several solid tumours, including ovarian cancer (11). On the other hand, mutations in SAMHD1 are linked to a severe congenital autoinflammatory disease known as Aicardi–Goutières syndrome (AGS) characterized by a dysregulated interferon (IFN) signalling due to defects in self and nonself nucleic acids recognition (12). Indeed, SAMHD1 has been recently proposed as a key regulator of cellular RNA homeostasis, demonstrating a relevant role in the recognition and buffering of immunogenic self RNAs, a process that regulates innate immune responses (13–15). Moreover, SAMHD1 has also been linked to DNA damage response, suggesting that it can influence anticancer therapy following DNA damage induction (16). Thus, here we evaluated the role of SAMHD1 expression and function in ovarian cancer *in vitro* and in patient cohorts.

## Materials and methods

### Cell lines and generation of *SAMHD1* knock-down cells

Human OVCAR3 cells were obtained from American Type Culture Collection (ATCC) and grown in RPMI 1640 medium supplemented with 10% heat-inactivated fetal bovine serum (FBS; ThermoFisher), 100 U/mL penicillin, 100 μg/mL streptomycin (Life Technologies) and insulin solution human (0.01 mg/mL) (Sigma-Aldrich) and maintained at 37°C in a 5% CO_2_ incubator. Human SKOV3 cells were obtained from ATCC and grown in Gibco™ McCoy’s 5A (Modified) Medium (ThermoFisher) supplemented with 10% of heat-inactivated fetal bovine serum (FBS, ThermoFisher) and antibiotics (100 U/mL penicillin, 100 μg/mL streptomycin (Life Technologies)) and maintained at 37°C in a 5% CO_2_ incubator.

Ovarian cancer cells were transfected following standard procedures. In brief, siRNAs targeting SAMHD1 gene (siSAMHD1, L-013950-01, ON-TARGETplus Human SAMHD1 siRNA Smartpool, Dharmacon, Cultek, Spain) were mixed with Lipofectamine 3000 reagent (ThermoFisher) at a final concentration of 100 nM and let stand for 20 min. Then, lipofectamine-siRNA complexes were mixed with 1.6x10^5^ cells and seeded in 24-well plates in the absence of serum, using OPTIMEM medium (Invitrogen). After 24h, medium with serum was added and left untreated 24h more. 48h after transfection, downregulation of gene expression was assessed at RNA (1x10^5^ cells were used) and protein (8x10^5^ cells were used) level. A non-targeting siRNA (siNT, D-001810-10, ON-TARGETplus Non-targeting Pool, Dharmacon, Cultek) was used as a control for putative off-targets effects in all experiments.

### Generation of SAMHD1 knock-out cell lines

T47D cells were obtained from Sigma-Aldrich-ECACC (European Collection of Authenticated cell cultures, 85102201-1VL) and grown in complete Dulbecco’s modified Eagle’s medium (DMEM, ThermoFischer) supplemented with 10% of heat-inactivated fetal bovine serum (FBS, ThermoFisher) and antibiotics 100 U/ml penicillin, 100 μg/ml streptomycin (Life Technologies) and maintained at 37°C in a 5% CO_2_ incubator. For generation of *knock-out* (KO) cells, T47D cells were transfected with a plasmid expressing a CRISPR-Cas9 construct designed to disrupt the sequence corresponding to exon 5 of *SAMHD1* gene that encodes for HD domain (CRISPR-SAMHD1), as described previously (11).

### Drugs and *in vitro* treatment

OVCAR3 cells were treated at indicated doses with the corresponding drugs for 24 hours or left untreated as a control. Lipopolisaccaride (LPS) and Polyinosinic–polycytidylic acid (Poly(I:C) were purchased from Sigma-Aldrich (L2630-10MG and P1530-25MG, respectively). Carboplatin was obtained from ThermoFisher (J60433.06).

### RNA-Sequencing and library preparation

Cellular RNA was extracted from cells using the NucleoSpin RNA II kit (Magerey-Nagel), as recommended by the manufacturer, including the DNase I treatment step. RNA-sequencing samples were prepared in biological duplicates. After quality control check, RNA library was constructed using Illumina TruSeq Stranded mRNA LT Sample Prep Kit and sequencing was performed using NovaSeq 6000 System with 150 bp paired ends reads (Macrogen). Sequencing files can be accessed on gene expression omnibus repository (GSE215309).

### Transcriptomic analysis

Transcriptomic analysis was performed as implemented in the computational workflow for the detection of differentially expressed genes and pathways from RNA-seq data (17). Reads were aligned to the human GRCh38 (annotation NCBI_109.20200522) using HISAT2. Low-expression genes with at least one zero counts were filtered out and the remaining reads normalized with Relative Log Expression (RLE) method as implemented in DESeq2 R library. Differential gene expression between the control and treatment groups was estimated with the DESeq2 Wald test. Sequencing files can be accessed on gene expression omnibus repository (GSE215309).

Gene Set Enrichment Analysis (GSEA) was performed on a pre-ranked GSEA list based on Log 2FC values of differentially expressed genes (DEGs: Log2FC > 1, p-value < 0.05), against Molecular Signatures Database (MsigDB v7.4) “Reactome” gene-set. Weighted enrichment statistics were based on 1000 permutations. Significantly enriched gene-sets with FDR adjusted q-value<0.1 were selected for Enrichment map visualization as previously described. Briefly, enrichment files were inputted into the Enrichment Map app within the Cytoscape program for visualization. Parameters were set at default values (node cutoff FDR Q value 0.1; Jaccard Overlap combined coefficient cutoff 0.375, k-constant 0.5). Nodes were manually laid out and combined into a common biological process for clarity using the AutoAnnotate app.

### Human cytokine network array

Total RNA was extracted using the NucleoSpin RNA II kit (Macherey-Nagel), as recommended by the manufacturer, including the DNase I treatment step. Reverse transcription was performed using the PrimeScript™ RT-PCR Kit (Takara) following manufacturer instructions. Cytokine expression was evaluated by using the commercial TaqMan Human Cytokine Network array (4414124, ThermoFisher) which included primers and probes for 28 cytokine network associated genes and 4 endogenous control genes. Relative expression of the distinct cytokine genes was measured by two-step quantitative RT-PCR and normalized to *GAPDH* expression by using the DDCt method.

### Quantitative RT-polymerase chain reaction

Total RNA was extracted and reverse transcribed as above described. mRNA levels of all genes were measured by two-step quantitative RT-PCR and normalized to *GAPDH* mRNA expression using the DDCt method. Primers and DNA probes were TaqMan Gene expression assays from Life Technologies (*DDX58*, TaqMan Hs01061436_m1; *IFIH1*, TaqMan Hs00223420_m1; *MB21D1* TaqMan Hs00403553_m1; *TMEM173* TaqMan Hs00736955_g1; *IL6*, TaqMan Hs00174131_m1; *SAMHD1*, TaqMan Hs00174103_m1; *IL8*, TaqMan Hs00174103_m1; *TNF*, TaqMan Hs00174128_m1; *IL18*, TaqMan Hs01038788_m1; *CXCL10*, TaqMan Hs00171042; *ISG15* TaqMan Hs00192713_m1).

### Western blot analysis

Cells were rinsed in ice-cold phosphate-buffered saline (PBS) and extracts were prepared in lysis buffer (50 mM Tris HCl pH 7.5, 1 mM EDTA, 1 mM EGTA, 1 mM NaV_3_O_4_, 10 mM sodium β-glycerophosphate, 50 mM NaF, 5 mM sodium pyrophosphate, 270 mM sucrose and 1% Triton X-100) supplemented with protease inhibitor cocktail (Roche) and 1 mM phenylmethylsulfonyl fluoride. Samples were electrophoresed in SDS-polyacrylamide gels and blotted onto nitrocellulose membranes. Blocked membranes were incubated overnight at 4°C with the following antibodies: anti-human Hsp90 (1:10000; 610418, BD Biosciences); anti-human GAPDH (1:10000; ab9485, Abcam); anti-SAMHD1 (1:2000; ab67820, Abcam); anti-Cleaved PARP1 (E51) (1:1000; ab32064, Abcam); anti-Cleaved caspase 3 (D175) (5A1E) (1:1000; 9664, Cell Signaling); anti-IRF7 (1:1000; 4920; Cell Signaling); anti-MDA5 (1:1000; 5321; Cell Signaling); anti-RIG-I (1:1000; 3743; Cell Signaling); anti-phosphoSTAT1 (Y701) (1:1000; 9167, Cell Signaling); anti-IFITM2 (1:1000; 13530, Cell Signaling); anti-β-Actin (1:1000; A5441, Sigma). After washing, the membranes were incubated with a secondary conjugated horseradish peroxidase-conjugated secondary antibodies for 1h at room temperature and then revealed with SuperSignal West Pico Chemiluminescent Substrate (Pierce Chemical).

### Patient cohort

A cohort of 22 ovarian cancer patients was collected from the Medical Oncology department of our hospital. Ovarian cancer diagnosis occurred between 1991 and 2018. All patients had been primarily treated with debulking surgery plus platinum-based chemotherapy. The study was conducted under the ethics principles of the Declaration of Helsinki and approved by the Research and Ethics Committee of Hospital Germans Trias i Pujol. Samples were obtained from the Biobank of the Institut d’Investigació Germans Trias i Pujol. All patients provided written informed consent.

*Variables studied:* age at diagnosis, histologic subtype (high grade serous *versus* others), *BRCA* genes status (pathologically mutated *versus* wild type, variants of unknown significance or unknown), SAMHD1 immunochemistry (positivity defined by cellular positivity 25%, as previously performed in AML cancers (11), progression free survival (PFS, defined as the time between first treatment and progression or death, whatever occurs first), overall survival (OS, defined as time from diagnosis to death from any cause).

### Construction of tissue microarrays and immunohistochemistry

TMA were constructed using a TMA workstation MTA-1 (Beecher instruments) and 3 different areas/tumor were selected and included (cylinders of 0.6 mm in diameter of each block of paraffin-embedded tissue). Then, TMA was cut in 5 micrometers sections for subsequent analysis. SAMHD1 expression was evaluated by immunohistochemistry (1:200, polyclonal rabbit anti-SAMHD1 antibody, cat. no. 12586-1-AP, Proteintech) in an automated detection system (Ultraview, Ventana 9 after antigen retrieval), as previously reported (11). Evaluation of SAMHD1 expression in the TMA sections was performed blinded by experienced pathologists and the percentage of SAMHD1 positive tumor cells was recorded. Independent triplicate evaluations were performed for each tumor. Then, tumors were classified as SAMHD1 positive or negative, depending on the percentage of SAMHD1 stained cells, which was arbitrarily defined, being SAMHD1 positive cases those with cellular positivity ≥25%, as previously performed (11). Histopathological unit of Hospital Germans Trias i Pujol performed all the immunohistochemical analyses.

### Statistical analysis

Experimental data were analysed with the PRISM statistical package and expressed as mean ± SD of at least three independent experiments performed in duplicate. *p*-values were calculated using an unpaired, two-tailed, t-student test.

Clinical variables were analysed using the SPSS statistical package. Descriptive statistics were medians and percentages, as appropriate. Correlation of SAMHD1 immunostaining positivity with clinical characteristics was studied with the *Pearson Chi-square test* (2-tailed). Median times for PFS and OS were estimated using the Kaplan–Meier method and reported with their confidence intervals (CI) at the 95% level. Log rank was used to compare Kaplan–Meier Curves. Cox analyses were used to estimate the effect of different variables on survival outcomes (hazard ratios).

## Results

**Figure 1:**
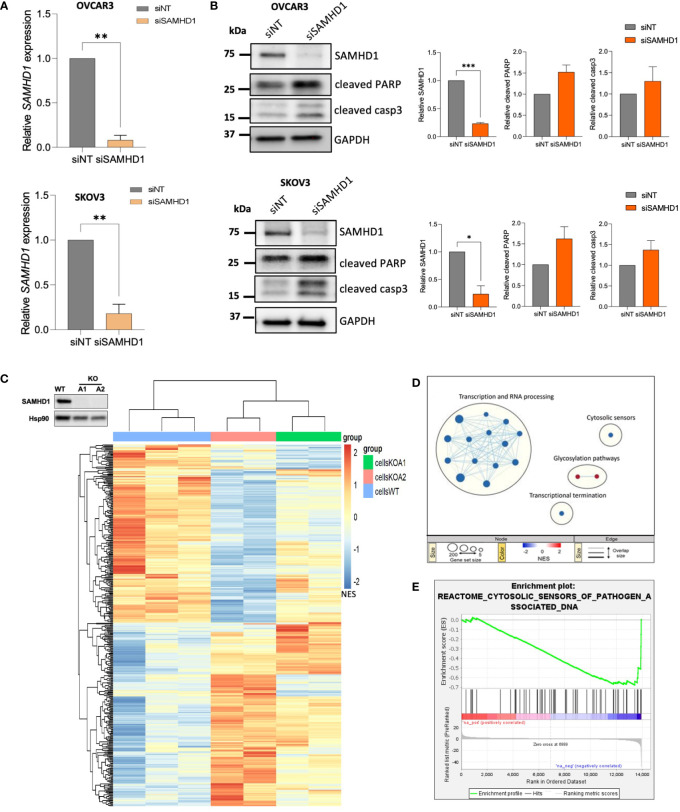
Depletion of SAMHD1 enhances apoptosis and regulates innate immune response. **(A)** Effective SAMHD1 downregulation by RNA interference in ovarian cancer cell lines. siRNAs targeting SAMHD1 gene (siSAMHD1) were transfected into SKOV3 cells (up) and OVCAR-3 cells (bottom). Gene expression was evaluated by RT-qPCR. **(B)** SAMHD1 knockdown induces apoptosis in ovarian cancer cells. Expression of apoptotic markers, cleaved PARP and cleaved Caspase 3 proteins, was measured by western blot in siRNA treated OVCAR3 (upper panel) and SKOV3 (bottom panel) cell lines. Representative western blot (left) and quantification (right) showing specific siRNA-mediated inhibition of SAMHD1 and increased cleaved PARP and cleaved caspase 3 expression. **(C)** Heatmap representation of gene expression changes in SAMHD1-KO and SAMHD1-WT cells. Heatmap was generated by unsupervised hierarchical clustering of significantly expressed genes (normalized enrichment score NES; p < 0.05). **(D)** Reactome Gene set enrichment map of significantly enriched pathways for SAMHD1-KO cells. Reactome Gene set clusters are annotated, and nodes manually laid out for clarity. Node size represents number of genes, node color represents significance (NES), and edge thickness represents number of shared genes. **(E)** Enrichment plot of the reactome cytosolic sensors of pathogen associated DNA geneset. Profile of the running ES Score of this geneset supports downregulation signature of cytosolic sensors and signalling pathways. Significantly down- or up-regulated genes **(C)** and gene sets **(D)** are highlighted in blue or red, respectively. *p<0.05; **p<0.005; ***p<0.0005.

To determine the contribution of SAMHD1 in ovarian cancer, we effectively downregulated SAMHD1 expression in the ovarian cancer cell lines OVCAR3 and SKOV3, leading to a 70-80% reduction in SAMHD1 RNA and protein expression levels (**Figures 1A, B**). We have previously shown that SAMHD1 knockout breast cancer cells presented increased DNA damage and apoptosis, an effect that was enhanced upon platinum-based treatment (11). In SKOV3 and OVCAR3 ovarian cancer cell lines, SAMHD1-depleted cells also showed increased expression of the apoptotic markers, cleaved PARP and cleaved caspase-3 expression, although differences were not statistically significant (**Figure 1B**), indicating the existence of additional mechanisms in contrast to previous data (11).

To investigate the signalling pathways affected by SAMHD1 depletion, we took advantage of SAMHD1-KO breast cancer cell lines, previously developed in our group (11), to perform a whole transcriptome profiling (**Figure 1C**; **Supplementary Figure 1**). Hierarchical clustering of wild-type and SAMHD1-KO cell clones using the union of all differentially expressed genes (DEGs) revealed distinct genetic signatures among them, while distinct knock-out clones presented more similar signatures, compared to wild-type (**Figure 1C**). To identify pathways specifically affected by the downregulation of SAMHD1 in breast cancer cells, we performed gene-set enrichment analysis (GSEA) using the Reactome gene-sets (**Figure 1D**). Overall, SAMHD1 knockout induced a global downregulation of several signalling pathways, especially at transcription and RNA processing level. More interestingly, downregulation of the cytosolic sensors of pathogen associated molecular patterns (PAMPs) was also observed in SAMHD1-KO cells, suggesting that downregulation of SAMHD1 may influence immune signalling and response *in vitro* (**Figures 1D, E**). Although transcriptomic data may vary between cell types, our results in breast cancer cells are also in accordance with previous transcriptomic data from monocytic cells where SAMHD1-KO clones showed dysregulation of several immune signalling pathways, such as RIG-I like receptors, IFN and cytokine signalling pathways (18) and thus, prompted the evaluation of similar pathways in ovarian cancer models.

**Figure 2:**
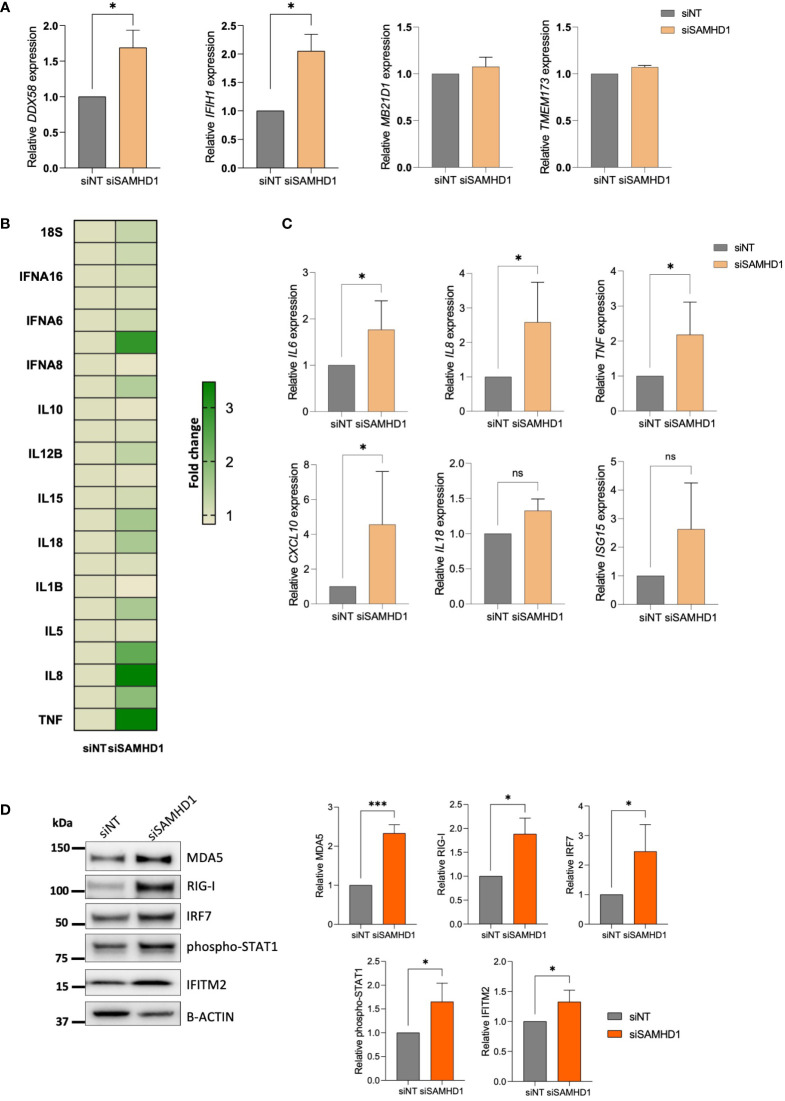
SAMHD1 knockdown modulates RLR (RIG-I like receptor) expression and innate immune signaling. **(A)** Gene expression of DNA sensors (*MB21D1* encoding cGAS protein, *TMEM173* encoding STING protein) and RNA sensors (*DDX58* encoding RIG-I protein, *IFIH1* encoding MDA5 protein), upon SAMHD1 knock-down. **(B)** SAMHD1 knockdown induces proinflammatory cytokine expression. Heatmap showing fold change increase expression in siSAMHD1 cells compared to non-targeting control, evaluated using the TaqMan Human Cytokine Network array. **(C)** Gene expression of distinct IFN-stimulated genes (ISG) in SAMHD1 knockdown cells. Increased *IL6*, *IL8*, *TNF*, *IL18*, *CXCL10* and *ISG15* expression upon SAMHD1 depletion was confirmed in additional experiments. **(D)** Representative western blot (left) and quantification (right) showing increased protein expression of distinct IFN-stimulated proteins in SAMHD1 knockdown cells. Protein expression of RNA sensors MDA5 and RIG-I, transcription factor IRF7, phosphorylation of STAT1 and IFITM2 was determined by western blot. Mean +/- SD of at least three independent experiments is shown. *p<0.05; ***p<0.0001. siNT, non-targeting siRNA used as control; siSAMHD1, siRNA specifically targeting SAMHD1. ns, non-significant.

Considering the key role of SAMHD1 in the induction of IFN-mediated immune activation derived from its role in DNA damage repair (16, 19), together with reported deficiencies in nucleic acid sensing and subsequent loss of innate immune activation in ovarian cancer (20), we focused our attention on innate immune response and cytosolic pattern recognition receptors (PRRs). First, we evaluated changes in the most common RNA and DNA PRR (**Figure 2A**). Interestingly, significant upregulation of both RNA sensors RIG-I, encoded by the *DDX58* gene and MDA5, encoded by the *IFIH1* gene was observed in siSAMHD1 cells, whereas DNA sensors cGAS (*MB21D1*) and STING (*TMEM173*) expression did not change upon SAMHD1 downregulation (**Figure 2A**). In addition, when ovarian cancer cells were exposed to LPS, known to recognize and activate TLR, or Poly(I:C), known to activate the cytosolic RNA helicases as RIG-I and MDA-5 (21), induction of IFN-stimulated genes (ISG) was only observed with poly(I:C) treatment, further supporting the more prominent role of RNA sensors in IFN-mediated response in ovarian cancer cells (**Supplementary Figure 2**). Then, we evaluated a comprehensive set of 28 cytokine associated genes included in the commercial TaqMan Human Cytokine Network array, finding significant transcriptional changes associated to SAMHD1 depletion in *IFNA7*, *IFNB1*, *IL16*, *IL18*, *IL4*, *IL6*, *IL8*, *LTA* and *TNF* (**Figure 2B**). These findings were confirmed in independent experiments, indicating an increased IFN-induced signalling upon SAMHD1 downregulation (*IL6 p*=0.0259, *IL8 p*=0.0173 and *TNF p*=0.023, respectively) (**Figure 2C**, upper panel). Interestingly, further evaluation of additional innate immune activation pathways showed similar results, i.e., expression of IFN-stimulated genes (ISG) as *CXCL10* (*p*=0.0327) and *ISG15* were also upregulated in SAMHD1-depleted cells (**Figure 2C**, bottom panel) and SAMHD1 knockdown also induced increased protein expression of the PRR, MDA5 and RIG-I, IRF7 transcription factor and IFN-induced transmembrane protein IFITM2 as well as enhanced phosphorylation of STAT1 (**Figure 2D**), all suggestive of enhanced IFN-mediated inflammation.

**Figure 3:**
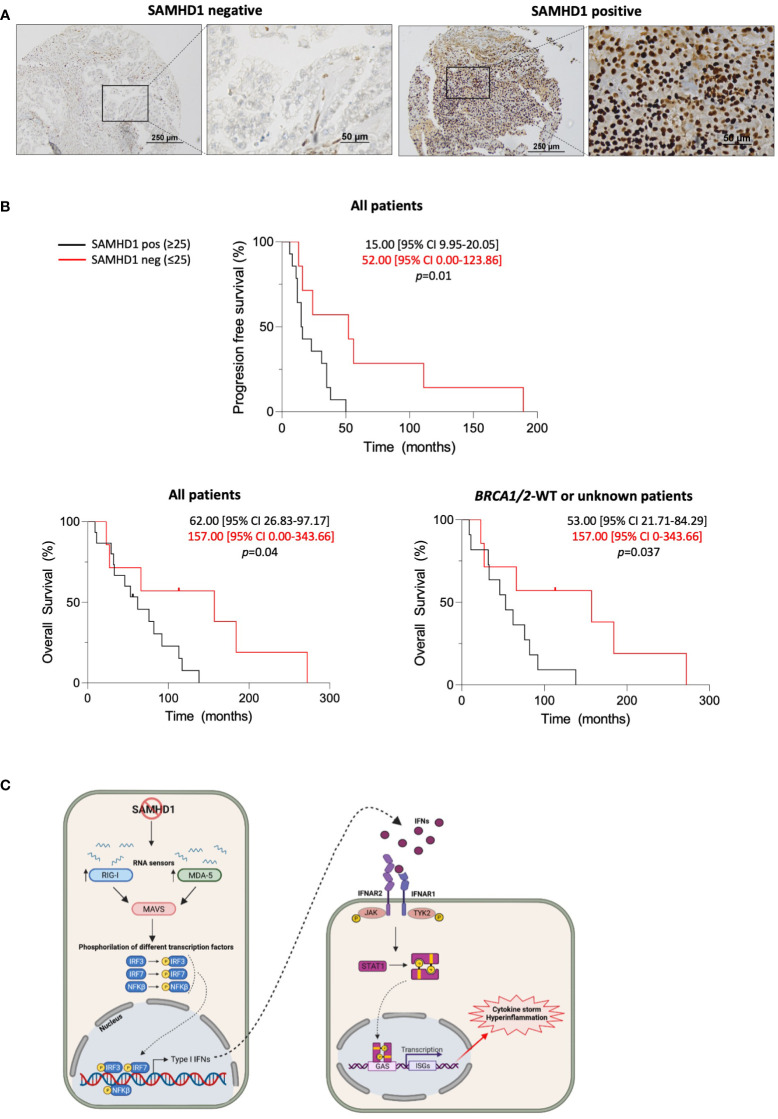
SAMHD1 expression is a prognostic factor in ovarian cancer patients. **(A)** Representative microscopy images of SAMHD1 expression in paraffin-embedded ovarian tumour biopsies. Images on the left represent SAMHD1-low expressing tumours and on the right positive expressing tumours. SAMHD1 expression was evaluated by immunohistochemistry in tumour samples. High expression of SAMHD1 observed in lymphocytic cells infiltrating in the tumours was used as a positive control of immunohistochemistry for negative or low expressing biopsies. **(B)** Kaplan-Meier curves of progression-free survival (PFS, top) and overall survival (OS, bottom) stratified according to SAMHD1 status, i.e, SAMHD1-low (SAMHD1 expression below 25% in cancer cells, red lines) or SAMHD1-high (SAMHD1 expression equal or above 25%, black lines). Median survival times with 95% CI of both groups are shown. Log rank test was used to test the significance and censored patients are indicated by a vertical line. **(C)** Working model of the innate immune signalling pathways triggered by the absence or the low expression of SAMHD1 in ovarian cancer. Low SAMHD1 expression induces increased levels of distinct IFN-stimulated genes in ovarian cancer cells, subsequently leading to increased antitumoral immunity and better prognosis in patients. Abbreviations are as follows: MDA5, melanoma differentiation-associated protein 5; RIG-I, retinoic acid-inducible gene-I; IFN, interferon; IFNAR, interferon a/b receptor 1; JAK, Janus kinase 1; TYK2, tyrosine kinase 2; STAT, signal transducer and activator of transcription; P, phosphoryl group; ISG, interferon-stimulated genes; IL6, Interleukin 6; IL8, Interleukin 8; TNF, Tumour Necrosis Factor; CXCL10, C-X-C motif chemokine ligand 10. Created with BioRender.

In view of these findings, the role of SAMHD1 was also evaluated in a cohort of 22 ovarian cancer patients, previously described (11). Median age at diagnosis was 63.00 years (min-max 51-82 years), the most frequent histology was high-grade serous subtype (n=17, 77.3%), and 4 patients were known to harbour germline pathologic *BRCA1/2* mutations (18.2%, all of them with high-grade serous histology). Median progression free survival (PFS) and median overall survival (OS) of the whole sample were 16.00 months (95% CI 5.66-26.34), and 66.00 months (95% CI 33.03-98.96), respectively (**Table 1**). SAMHD1 expression was re-evaluated by immunohistochemistry in ovarian cancer archival biopsies (**Figure 3A**), that were classified as SAMHD1 positive or negative depending on the percentage of SAMHD1 stained tumoral cells [positivity was arbitrarily defined as those with cellular positivity ≥25%, based on previous reported thresholds (11)]. As previously reported, SAMHD1 positivity correlated with high-grade serous histology (*p*=0.007), but not with *BRCA1/2* status (*p*=0.144). More importantly, SAMHD1 expression showed a statistically significant effect on survival outcomes. Median progression free survival (PFS) of the SAMHD1-high expression subgroup was statistically significantly shorter than those of the SAMHD1-low expression subgroup (15.00 [95% CI 9.95-20.05] *vs.* 52.00 [95% CI 0.00-123.86], *p*=0.010) (**Figure 3B**, upper panel). Median overall survival (OS) of the SAMHD1-high expression subgroup was also shorter than those of the SAMHD1-low expression subgroup (62.00 [95% CI 26.83-97.17] *vs.* 157.00 [95% CI 0.00-343.66], *p* 0.040) (**Figure 3B**, bottom panels). Hazard ratio for PFS was 4.54 (95% CI 1.27-16.23, *p*=0.020), and hazard ratio for OS was 3.564 (95% CI 0.99-12.56, *p*=0.052), favouring the low-expression subgroup. These differences remained statistically significant when individually analysing the *BRCA* wild type or unknown subgroup (**Figure 3B**, lower panel).

**Table 1:** Baseline characteristics of the clinical sample and survival outcomes.

	Descriptive statistics (%)	Median PFS (months, 95%CI)	*p-value*	Median OS (months, 95%CI)	*p-value*
**Age at diagnosis** (years)	63 (51-82 years)	NA		NA	–
**Histologic subtype**			0.040		0.144
High grade serous Others*	17 (77.30%) 5 (22.70%)	16.00 (13.02-18.98) 52.00 (0.00-112.12)		66.00 (37.59-94.40) 157.00 (0-359.77)	
**BRCA status**			0.372		0.857
Mut** Wild type/UK***	4 (18.20%) 18 (81.80%)	12.00 (4.16-19.84) 23.00 (6.37-39.63)		113.00 (0-238.75) 62.00 (34.97-89.03)	
**SAMHD1**			0.010		0.040
<25 >25	7 (31.80%) 15 (68.20%)	52.00 (0.00-123.86) 15.00 (9.95-20.05)		157.00 (0.00-343.66) 62.00 (26.83-97.17)	
**Total**	22	16.00 (5.66-26.34)		66.00 (33.03-98.96)	–

*4 clear cells and 1 low grade serous tumour; **Pathologically mutated; *** UK, unknown. NA, not-applicable.

The contribution of the observed differences in PFS and OS depending on SAMHD1 expression might be partially affected by previous platinum-based chemotherapy which was common to all patient cohort. However, *in vitro* exposure to carboplatin did not induce a differential response in SAMHD1 knock-down cells, compared to non-targeting control (**Supplementary Figure 3**), suggesting that SAMHD1 expression might be the main contributor to the observed correlation with low-SAMHD1 and better survival outcomes.

Overall, clinical data allowed us to propose SAMHD1 as a prognostic marker in ovarian cancer, whose function might putatively induce antitumoral innate immune response, as demonstrated *in vitro* in cell lines (**Figure 3C**). Interestingly, exploring the ovarian cancer proteome using TCGA transcriptomics data obtained from Human Protein Atlas database (www.proteinatlas.org) (22), we observed that high expression of several innate immune activation hallmark genes was associated to improved ovarian cancer survival, supporting the idea that upregulation of innate immune response is linked to better prognosis in ovarian cancer, a mechanism that is regulated by SAMHD1, as demonstrated *in vitro* (**Supplementary Figure 4**).

## Discussion

Ovarian cancer (OC) is the third most diagnosed gynaecologic cancer in Europe (23). However, despite the recent advances in surgery and chemotherapy, it remains the most lethal gynaecologic cancer. In this context, a better understanding of the induction and modulation of the anti-tumor innate immunity is key to develop new therapies. Here, we provide evidence of the involvement of SAMHD1 in the induction and modulation of anti-tumoral immunity in ovarian cancer.

Our evaluation of innate immune activation pathways revealed an increased IFN-induced signalling upon SAMHD1 downregulation, concomitant with an upregulation of MDA5 and RIG-I RNA sensors. Indeed, in agreement with our data, SAMHD1-deficiency and subsequent accumulation of endogenous RNA substrates is a cause of type I interferonopathies, characterized by an upregulation of distinct IFN-stimulated genes (ISGs) (20, 24). Moreover, it has also been shown that chronic interferon response in SAMHD1-KO mice was driven by the MDA5 pathway in close concordance with our data in ovarian cancer cells (25) and further supporting the idea that SAMHD1 depletion is able to enhance innate immune activation and inflammation in cancer cells, a process that might have an important impact on ovarian cancer clinical outcome. In view of the promising pre-clinical data, we evaluated the role of SAMHD1 in 22 ovarian cancer patients. Interestingly, SAMHD1 expression was significantly associated with tumor histology, being high-grade serous histology ovarian tumors those presenting the highest proportion of SAMHD1, as previously reported in other cancer types (11). Although no significant association between SAMHD1 and *BRCA1/2* status was found, all *BRCA1/2* mutated patients showed high expression of SAMHD1, suggesting a correlation between these two variables. In this sense, it has been described that unprotected stalled replication forks in SAMHD1-deficient cells can produce cytoplasmic DNA (26), which could mimic DNA fragments generated from stalled replication forks in *BRCA1/2*-mutant cells during the S-phase or DNA damage repair. Thus, high expression of SAMHD1 could represent a compensatory mechanism in response to the inflammatory response associated with the release of cytosolic DNA as a consequence of defective DNA repair secondary to *BRCA1/2* mutations. More interestingly, SAMHD1 positivity was significantly associated with poorer prognostic clinical outcomes, including decreased median progression free survival (PFS) and median overall survival (OS). Overall, clinical data allow us to propose SAMHD1 as a prognostic marker in ovarian cancer, whose function might putatively induce antitumoral proinflammatory innate immune response, as demonstrated *in vitro* in cell lines. Interestingly, exploring the ovarian cancer proteome using TCGA transcriptomics data obtained from Human Protein Atlas database (www.proteinatlas.org) (22), we observed that high expression of several innate immune activation hallmark genes was associated to improved ovarian cancer survival, supporting the idea that upregulation of innate immune response is linked to better prognosis in ovarian cancer, a mechanism that is regulated by SAMHD1, as demonstrated *in vitro* (**Supplementary Figure 4**).

Although inflammation and cancer onset and progression are closely interrelated, in ovarian tissue, inflammation is a double-edge sword that has been associated with either tumour progression or suppression (27, 28), highlighting the importance of characterizing specific inflammatory pathways. Along the same line, increasing amounts of data are pointing towards the importance of nucleic acid-sensing pathways in cancer patients progression (29). In concordance with our data, several evidences indicate the capability of tumour cells to generate inflammatory factors, representing key signals that determine the cross-talk between tumour and immune cells and ultimately affect the mechanisms of immunosuppression by which tumour cells circumvent innate and adaptive immune responses (30). In our study, we show that depletion of SAMHD1 in ovarian cancer cells leads to upregulation of RNA helicases and also several IFN-stimulated genes, as cytokines and chemokines, suggesting an activation of innate immune signalling pathways that could trigger an inflammatory response in the tumour site (**Figure 2C**) and may ultimately affect patient prognosis. In fact, it has been largely described that some cytokines can act as potent chemoattractant for different cell subsets; for example, IL6 and IL8 are direct mediators of T cell migration (31, 32). Moreover, the presence of T cells in ovarian tumours has been associated with a survival advantage in distinct studies across diverse patient cohorts (33). However, whether tumoral cells are capable of effectively initiating an antitumoral IFN-mediated response *in vivo* remains to be characterized.

In conclusion, our data provides evidence of the involvement of SAMHD1 in ovarian cancer, as previously reported in other cancer types (11). Next steps should aim to validate these results in larger series, as well as prospectively explore the correlation between SAMHD1 expression, innate immune response, and inflammatory chemokines directly in ovarian cancer patients. Moreover, given that the activation of innate immunity in response to the inactivation of SAMHD1 described in this work, together with the described innate immune response triggered by BRCA1/2 abrogation (34), we suggest that TILs recruitment processes could be exploited to develop potential novel immunotherapy treatment.

## Data availability statement

The data presented in the study are deposited in the omnibus repository repository, accession number GSE215309. Other original contributions presented in the study are included in the article/**Supplementary Material**. Further inquiries can be directed to the corresponding authors.

## Ethics statement

The studies involving human participants were reviewed and approved by Hospital Germans Trias i Pujol. The patients/participants provided their written informed consent to participate in this study.

## Author contributions

LG-C and EF were responsible of all experimental tasks, including biological analysis in cell lines and immunohistochemistry of patient samples and manuscript drafting. IJE performed RNAseq data analysis. IT, AM-C and MR were responsible of patient selection, clinical data collection and clinical data analysis. AB-P and ER-M helped in obtaining experimental data, contributed to data analysis and were responsible of methodological set-up. BC was responsible of study conception and writing review. MR, MM and EB were responsible of formal analysis, writing and review. EB and MM had substantial contribution to the conception, conceptualization, formal analysis, manuscript writing and final review. All authors contributed to the article and approved the submitted version.
