# Supplementary material for: SAMHD1 expression modulates innate immune activation and correlates with ovarian cancer prognosis
Source: Front Immunol. 2023 Feb 9;14:1112761. doi: 10.3389/fimmu.2023.1112761 (PMC9948397; doi:10.3389/fimmu.2023.1112761)

Supplementary information

Supplementary Figure 1

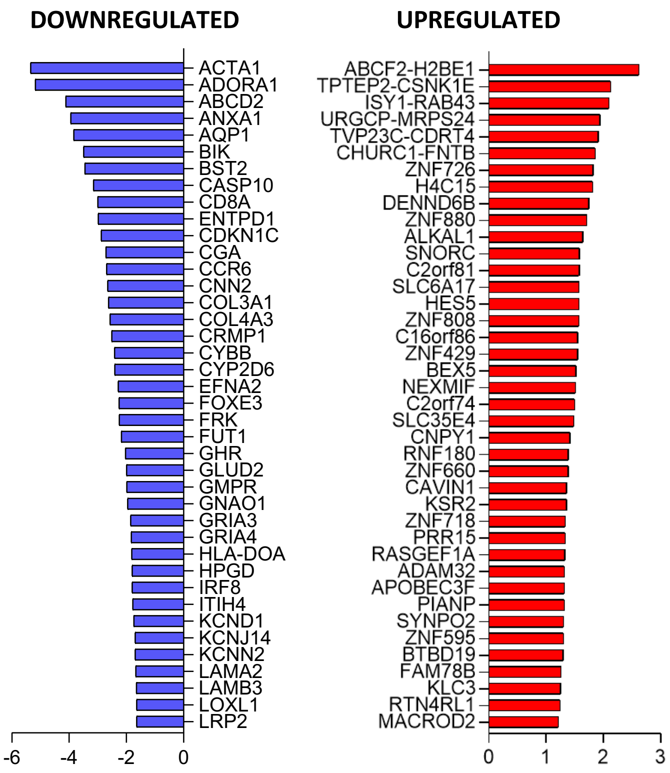

Supplementary figure 1. Bar plots of top 40 differentially enriched genes (DEG) for SAMHD1-KO breast cancer cells relative to SAMHD1-WT, based on Log2 gene expression (Log2FC) and p<0.05. Significantly down- or up-regulated DEG are highlighted in blue or red, respectively.

Supplementary Figure 2

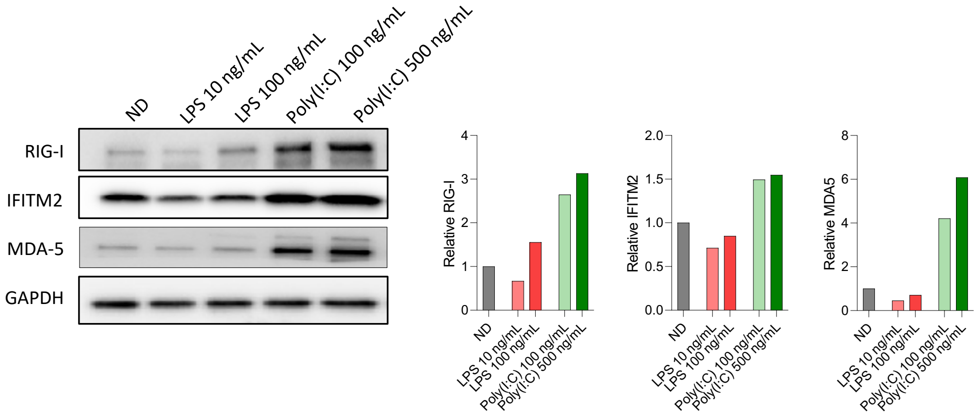

Supplementary figure 2. Expression of RNA sensors and ISG upon exposure to LPS and polyI:C in OVCAR cells. PolyI:C, but not LPS is reported to specifically activate the cytosolic RNA helicases retinoic acid-inducible protein I (RIG-I) and melanoma differentiation-associate gene 5 (MDA-5) (21). Indeed, in OVCAR cells, only polyI:C treatment, was able to induce expression of RNA sensors and IFN-stimulated genes as IFITM2, further supporting the role of RNA sensors in IFN-mediated response. A representative western blot and associated quantification is shown.

Supplementary Figure 3

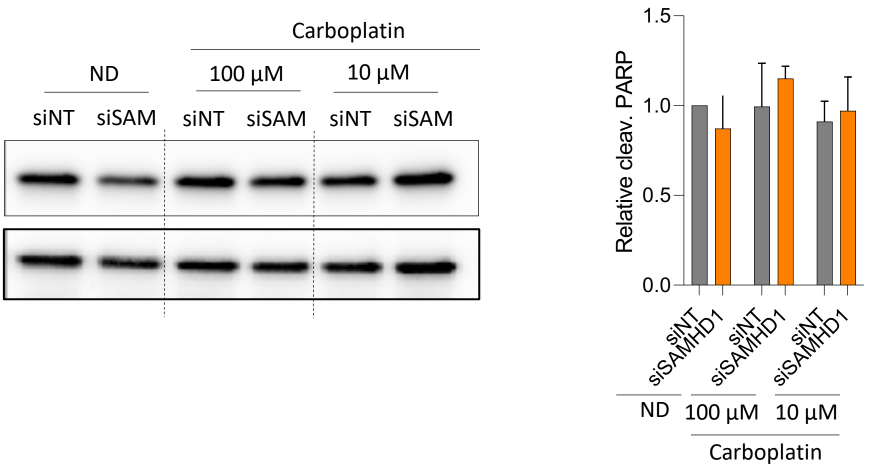

Supplementary figure 3. Carboplatin treatment did not differently impact expression of apoptotic markers in SAMHD1-depleted cells. Left, representative western blot analysis of PARP-cleaved expression upon carpoblatin treatment in SAMHD1 wt (siNT) or depleted (siSAMHD1) cells; right, band quantification of western blot analysis. Bars represent mean ± SD of three independent experiments.

Supplementary Figure 4

A. *CXCL10*

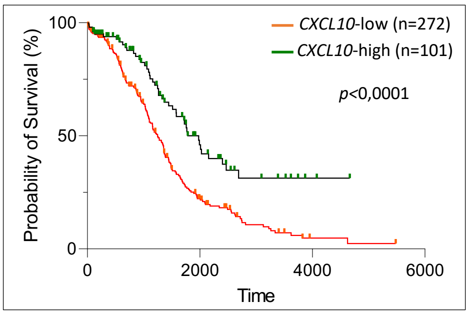

B. *ISG15*

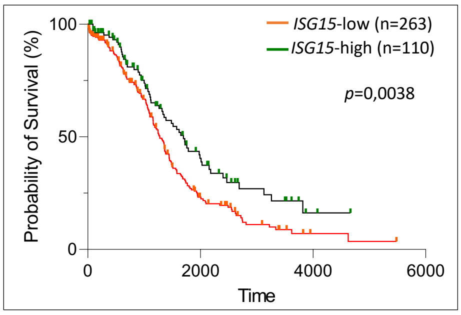

C. MDA5 (*IFIH1*)

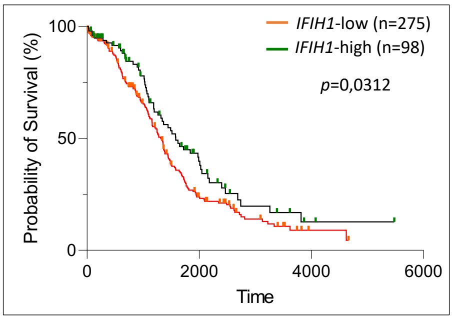

D. RIG-I (*DDX58*)

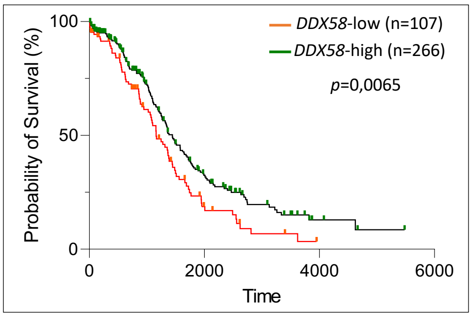

Supplementary figure 4. High expression of ISG whose expression is upregulated upon SAMHD1-depletion, is associated with improved ovarian cancer survival. Kaplan-Meier plots showing the correlation between mRNA expression of *CXCL10* (A), *ISG15* (B), MDA5-*IFIH1* (C) and RIG-I-*DDX58* (D) and ovarian cancer patient survival. Data was obtained from Human protein atlas (21) (www.proteinatlas.org) and patients were stratified according to best expression cut-off in high (green) or low (orange) expression group. Log rank p-values were calculated from analysis of the correlation between mRNA expression levels and patient survival.

Full length western blot to Fig 1B

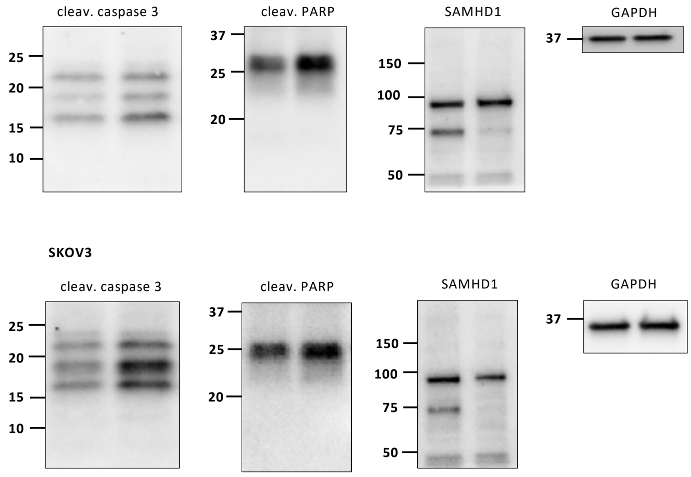

Full length western blot to Fig 2D

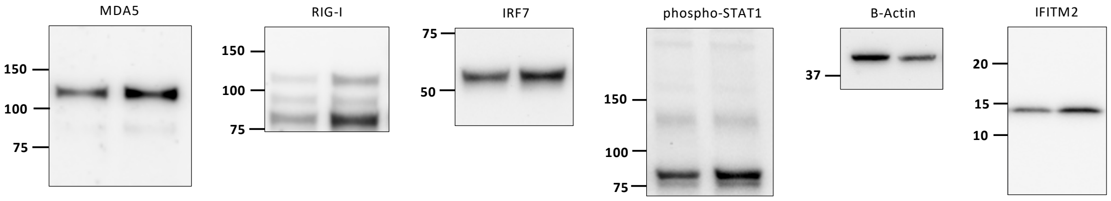

Full length western blot to Supplementary fig 1

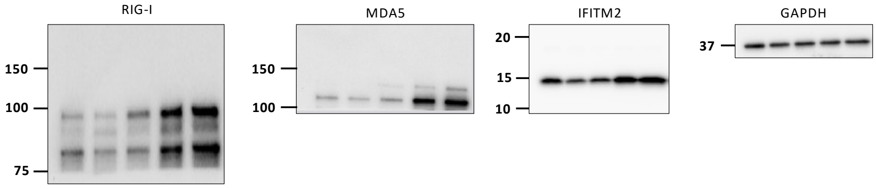

Full length western blot to Supplementary fig 2

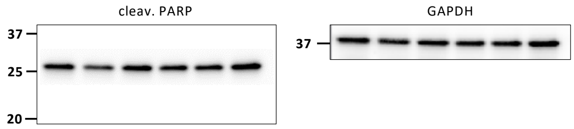

Supplement: Supplementary file 1 [file DataSheet_1.pdf]
